# “Why should I take drugs for your infection?”: outcomes of formative research on the use of HIV pre-exposure prophylaxis in Nigeria

**DOI:** 10.1186/s12889-015-1690-9

**Published:** 2015-04-10

**Authors:** John Idoko, Morenike Oluwatoyin Folayan, Nancin Yusufu Dadem, Grace Oluwatosin Kolawole, James Anenih, Emmanuel Alhassan

**Affiliations:** National Agency for the Control of AIDS, Abuja, Nigeria; Department of Child Dental Health, Obafemi Awolowo University, Ile-Ife, Nigeria; New HIV Vaccine and Microbicide Advocacy Society, Lagos, Nigeria; Institute of Public Health, Obafemi Awolowo University, Ile-Ife, Nigeria; Department of AIDS Prevention Initiative Nigeria (APIN), Jos University Teaching Hospital, Jos, Nigeria

**Keywords:** PrEP, Nigeria, Resistance, Stigma, Couples, Serodiscordant

## Abstract

**Background:**

Nigeria has the second highest number of new HIV infections annually. Therefore, it is important to explore new strategies for preventing new infections. The introduction of pre-exposure prophylaxis (PrEP) for use by persons at high risk of HIV infection has new potential in preventing new HIV infections. The aim of this study is to explore the public opinion, community interest, and perceptions about the use and access to PrEP in Nigeria.

**Methods:**

This formative study used a mixed method approach to collect data on public opinions and perceptions on appropriate target groups for PrEP access, community interest, perceptions about the use of PrEP as an HIV-prevention tool, how best to communicate with participants about PrEP, concerns about PrEP use by serodiscordant couples, and suggestions for the design and implementation of a PrEP demonstration project. Telephone and in-depth interviews were conducted, and focus group discussions and consultative meetings were held with critical stakeholders engaged in HIV-prevention, treatment, care, and support programmes in Nigeria. An online survey was also conducted.

**Results:**

HIV serodiscordant couples were identified as the appropriate target group for PrEP use. Most respondents felt that PrEP use by key affected populations would help reduce the HIV incidence. Stigma was identified as a major concern and a potential barrier for the acceptance and use of PrEP by HIV serodiscordant couples. Electronic and print media were identified as important means for massive public education to prevent stigma and create awareness about PrEP. In a male dominated society such as Nigeria, HIV-negative male partners in serodiscordant relationships may resist enrolment in PrEP programmes. This may be complicated by the fact that the identified index partner in most serodiscordant relationships in Nigeria is an HIV-positive woman, who is often diagnosed during pregnancy.

**Conclusions:**

PrEP uptake and use by HIV serodiscordant couples in Nigeria may face notable but surmountable challenges. Much depends on the appropriateness of actions taken by multiple players. Motivation of HIV-negative male partners to use PrEP and establishment of effective public education programmes in addressing stigma are essential.

## Background

Access to treatment for people living with HIV has improved in Nigeria in recent years. However, the pace of expansion remains slow and is far from meeting the universal target [1]. Nigeria has the second highest number of new HIV infections annually [2], and prevention efforts have yet to significantly slow the epidemic. Unless the influx of new infections is slowed, the Nigerian government will be less able to assist people in need, and AIDS will continue to devastate individuals, families, communities, and nations.

Owing to these concerns and the shift in the focus of HIV and AIDS management from a short-term emergency to a long-term concern, recent years have witnessed a renewed commitment by the Nigerian government to HIV prevention. This commitment is increasingly focused on developing new biomedical, behavioural, and structural approaches to preventing HIV transmission and implementing proven approaches in combination that are tailored to specific epidemiological and social contexts.

Large-scale clinical trials evaluating the use of antiretroviral drugs for HIV prevention have shown success [3-7] and elated the HIV-prevention field. Still, the impact of proven interventions may vary among locales because the HIV epidemic differs geographically. A 2010 antenatal sentinel survey in Nigeria showed a national HIV prevalence of 4.1%, ranging between 2.0% and 10.0% in various states. The HIV prevalence is generally higher in urban areas (4.8%) than in rural areas (2.6%), though the HIV prevalence is up to 21.3% in some rural areas [8]. Although the 2012 national HIV household survey showed a mean HIV prevalence of 3.1% [9], there are variations within the population. For instance, HIV prevalence among non-brothel based female sex workers (FSW) was 27.4%, compared with 17.2% in men who have sex with men (MSM) and 4.2% in people who inject drugs (PWID) [10].

Even within communities, the prevalence differs. For example, within the PWID community, women are seven to 33 times more likely to be HIV-positive than men [11]. Among diagnosed HIV serodiscordant couples in Nigeria [12-14], women have an approximately 11 times greater risk of being HIV-positive than HIV-negative [14]. The high incidence of HIV serodiscordance among couples increases the risk of HIV transmission to the HIV-negative partner unless concerted HIV-prevention efforts are taken.

Women and girls are particularly vulnerable as the existing HIV-prevention tools are not well suited for their control. Current economic hardships force many spouses to work away from home for long periods, and there is little guarantee that monogamy is reciprocated by the male partner in marital or stable relationships. Gender norms condoning multiple sexual partnerships for men exacerbate this and consequently increase women’s vulnerability. Furthermore, male and female condoms, which are the most commonly promoted risk-reduction strategy, require that male partners elect or agree to use them. This makes condoms potentially useless as an HIV-prevention strategy in situations of rape and sexual violence. Pre-exposure prophylaxis (PrEP) oral medication can be used by women–covertly if desired–making them an important addition to the HIV-prevention toolkit.

Considering these realities, it is important that Nigeria apply new combinations of proven and mutually reinforcing biomedical, behavioural, and structural interventions through an iterative process. These strategies include the use of PrEP, which holds promise in protecting many of the most vulnerable and at-risk populations, including the HIV-negative partners in serodiscordant partnerships, MSM, sex workers, women, and girls. Therefore, it is critical to explore the feasibility of PrEP use in combination with other prevention strategies to address the HIV epidemic in Nigeria, particularly in key target groups.

This formative study was conducted to explore public opinions on PrEP including community interest and perceptions its use as part of the HIV-prevention armamentarium in Nigeria. Specifically, this formative study sought to identify the appropriate study population for recruitment in a potential PrEP demonstration project by surveying public opinion, community interest, and perception about the use of PrEP as part of HIV prevention in Nigeria and to explore how best to discuss PrEP with various stakeholders before, during, and after the proposed PrEP demonstration project. This information will inform the design and implementation of a subsequent PrEP demonstration project in Nigeria.

A critical social ecological perspective was chosen for this study [15,16]. Social ecological perspectives posit that individual behaviours, such as intention to use and adhere to antiretrovirals (ARVs), are influenced by individual characteristics, interpersonal relationships, and macro-level factors. Individuals adapt themselves and their HIV-related behaviours to proximal and distal social contexts and structures, while simultaneously, these multiple social systems are affected by individual behaviours. We examined the potential of use of PrEP at both an individual (serostatus, attitudes, perceived efficacy) and structural (cultural norms, stigma, institutionalized discrimination, legal structures, policy environments) level.

## Methods

### Study design

This was an exploratory descriptive study and was not designed to test a hypothesis. The study objectives were devised assuming that understanding the factors effecting the adoption and use of PrEP as an HIV-prevention tool will help in designing effective programmes in communities targeted for intervention. We used a mixed method approach that included telephone interviews; in-depth interviews (IDIs); focus group discussions (FGDs); consultative meetings with critical stakeholders in HIV prevention, treatment, care, and support programmes in Nigeria; and an online survey to explore opinions on the design of a future PrEP demonstration project in Nigeria. This preliminary study was conducted between 30 October, 2013, and 4 February, 2014.

### Study setting

Quantitative studies were conducted in the proposed sites for a future PrEP clinical study as follows: Abuja (Federal Capital Territory), Edo, Cross River, and Benue states. Benue, Edo, and Cross-River states were proposed as sites for a future PrEP demonstration project owing to their high HIV prevalence [8]. Stakeholders engaged in the national HIV response in Nigeria primarily reside in Abuja; therefore, the state was also included as a study setting. Data were also collected from participants outside of these specific study sites through an online survey.

### Study participants

Key stakeholders aged 18 years older who were involved in the design and implementation of HIV-prevention and treatment research, programmes, and policies in Nigeria for at least 12 months were recruited to participate in the telephone and IDIs. Among eligible individuals were representatives of the Federal and State Ministry of Health, Federal Health Parastatals, and the National and State Agencies for the Control of AIDS (NACA); National HIV Prevention and Treatment Technical Working Group members; donor organizations and implementing partners; HIV-prevention and treatment researchers; ethicists and members of health research ethics committees; public health officials, health care providers, and administrators, and others involved in public health programmes; grassroots community leaders such as representatives of nongovernmental organisations (NGOs) and community-based organizations (CBOs) implementing HIV-prevention, care, and treatment programmes for people living with HIV, religious leaders, and other community group leaders (youth group leaders, community advocates, tertiary institutions); and other stakeholders such as journalists and religious leaders.

Participants recruited for the FGDs were representatives of NGO and CBO groups implementing HIV-prevention, care, and treatment programmes for people living with HIV, health care workers, people living with HIV, serodiscordant couples, and key affected populations (MSM, FSW, and PWID). Participation in the online survey was open to all interested persons worldwide.

### Data collection

#### Telephone interviews

A list of 238 persons working in the HIV and AIDS field as researchers, ethicists, journalists, implementing partners, development partners, policymakers, and health care providers was generated from the NACA programme office. Everyone on the mailing list was emailed brief background information on PrEP and a request to participate in a telephone interview by sharing their telephone number and a convenient date and time for the interview. Quantitative and qualitative data were collected during the interview using a guide comprising 11 questions. The second, third, fifth, and eleventh questions were open-ended to determine personal opinions and perspectives on PrEP including thoughts, concerns, and suggestions for implementation of a PrEP demonstration project. Respondents had the option to comment and further discuss any of the seven close-ended questions. Interviewers were also free to further explore any comments made by respondents during the telephone interview. The topics covered during the interview included the knowledge about PrEP, suggested target groups for the PrEP clinical study, potential positive outcomes of PrEP, barriers to PrEP access, and perceived challenges to PrEP use. Table 1 summarizes the checklist and open-ended question asked of respondents.

**Table 1:** **Telephone interview questions discussing the proposed PrEP demonstration project**

**No.**	**Question** ^**a**^
1	Have you heard of PrEP before now?
2	What do you know about PrEP?
3	How did you learn about PrEP?
4	Do you think there is any potential positive outcome if Nigeria includes PrEP as part of its HIV-prevention package? What are your concerns?
5	If at the end of the extensive community consultation process there is consensus that PrEP should be implemented in Nigeria, which community do you think should be targeted during the pilot project and why?
6	Do you think we will encounter challenges when discussing PrEP with religious leaders? Why? How do you think we can address this challenge?
7	Do you think we will encounter challenges when discussing PrEP with community leaders? Why? How do you think we can address this challenge?
8	Do you think we will encounter challenges discussing PrEP with PLHIV? Why? How do you think we can address this challenge?
9	Do you think we will encounter challenges discussing PrEP with the community at large? Please could you explain the reason for your perspective? How do you think we can address this challenge?
10	Do you think Nigeria may face challenges in the implementation of PrEP as a HIV-prevention method? Please could you explain the reasons for your response? How do you think we can address this challenge?
11	Finally, is there anything else you would like to share with me about PrEP such as your thoughts, suggestions, queries, concerns, and advice?

^a^For questions 1, 4, and 6–10, participants responded as follows: yes, no, or no response. Questions 2, 3, 5, and 11 were open-ended, and respondents were permitted to provide additional comments.

No., number; PrEP, pre-exposure prophylaxis; PLHIV, people living with HIV.

#### In-depth interviews

A total 111 IDIs were conducted. IDIs allowed exploration of issues raised during the telephone interviews from a personal perspective and captured the respondent’s experiences, opinions, and feelings regarding sensitive topics and specific, relevant personal events. A semi-structured guide was used to explore issues including the appropriate target populations for PrEP use, PrEP messaging to target populations and the general public, public policy required to support PrEP interventions, PrEP intervention management and decision-making, integration of PrEP into existing HIV-prevention programmes, and building the capacity for PrEP rollout. Interviews were conducted with public health officials, national- and state-level policy makers, health care providers and administrators, HIV development and implementing partners, religious leaders, researchers, and ethicists. Written consent was obtained from for all participants except for one participant, who initially preferred to give verbal consent and later signed the interview transcript confirming that it was duly representative of what was discussed during the interview.

#### Focus group discussions

Thirteen FGD transcripts were analysed. The FGDs were conducted with relevant stakeholders who will be directly or indirectly affected by a PrEP programme. Four FGDs were each held in Benue, Edo, and Cross River States and one FGD in Abuja. In each state, the first FGD included key populations, namely MSM, male and female sex workers, and PWID; the second FGD included health care providers; the third included serodiscordant couples and people living with HI;, and the fourth FGD included representatives from HIV programmes and key community gatekeepers such as religious leaders and journalists. The size of each FGD ranged between six and 12 persons. The sole FGD in Abuja included representatives from HIV programmes and key community gatekeepers.

The FGD queried respondents on appropriate target populations for PrEP; logistical barriers to PrEP access, possible facilitators of PrEP access, and requisites for PrEP use; cultural barriers, facilitators, and requisites for using PrEP; and the benefits and appropriate costs for PrEP. The FGDs used case scenarios to elicit discussion.

Before participating in the FGD, each individual gave written consent for study participation after details of the study were explained. Participants were allowed to ask clarifying questions. Refreshments were provided during the FGD, and participants were paid $12.50 each as transportation reimbursement.

#### Online survey

The online survey explored public opinions on potential target groups for a PrEP demonstration study in Nigeria, reasons for the choices, potential positive outcomes of PrEP, barriers and challenges to PrEP access, and means to address these challenges. The online survey tool was adapted from the telephone interview checklist. Question 5 on the original checklist shown in Table 1 was expanded into 12 sub-questions (Table 2).

**Table 2:** **Sub-questions forming the online survey following adaptation of the telephone interview guide**

**No.**	**Question** ^**a**^
5a	The Nigeria HIV-prevention programme considers HIV-negative partners in serodiscordant relationships a high-risk group for HIV infection. One of the many studies on PrEP showed a 75% reduction in the HIV incidence when a HIV-negative partner used PrEP. Would you consider serodiscordant couples a priority target group for the PrEP demonstration project in Nigeria?
5b	Please give the reason(s) for your response to question 5a.
5c	The Nigeria HIV-prevention programme considers men who have sex with other men (MSM) a high-risk group for HIV infection. One of the many studies on PrEP showed a 44% reduction in the HIV incidence when a HIV-negative MSM partner used PrEP. Would you consider MSM a priority target group for the PrEP demonstration project in Nigeria?
5d	Please give the reason(s) for your response to question 5c.
5e	The Nigeria HIV-prevention programme considers male and female sex workers a high-risk group for HIV infection. No studies have been conducted to evaluate the efficacy of PrEP in preventing HIV infection in this specific population. Would you consider male and female sex workers a priority target group for the PrEP demonstration project in Nigeria?
5f	Please give the reason(s) for your response to question 5e.
5 g	The Nigeria HIV-prevention programme considers people who inject drugs (PWID) a high-risk group for HIV infection. One of the many studies on PrEP showed a 49% reduction in the HIV incidence when a HIV-negative PWID used PrEP. Would you consider PWID a priority target group for the PrEP demonstration project in Nigeria?
5 h	Please the give reason(s) for your response to question 5 g.
5i	The Nigeria HIV-prevention programme considers young women age 21 to 24 a high risk group for HIV infection. One of the many studies on PrEP showed a 62% reduction in the HIV incidence in heterosexual couples. The study did not specifically focus on young women. However, it showed that women can equally benefit from PrEP. Would you consider young women a priority target group for the PrEP demonstration project in Nigeria?
5j	Please give the reason(s) for your response to question 5i.
5 k	If at the end of the extensive community consultation process there is consensus that PrEP should be implemented in Nigeria, which of the target populations that you have identified could benefit from PrEP do you think the country should focus on during the pilot project?
5 l	Please give the reason(s) for your response to question 5 k.

^a^Questions in the online survey were designed based on the responses to question #5 in the initial telephone interview.

No., number; PrEP, pre-exposure prophylaxis; MSM, men who have sex with men; PWID, people who inject drugs.

The online survey was administered by the following organizations: the New HIV Vaccine and Microbicide Advocacy Society listserv, which is a forum providing information on biomedical HIV-prevention research and development to 5,432 subscribers; International Rectal Microbicide Advocacy listserv, a forum providing information on biomedical HIV-prevention research and development focused on MSM and transgender issues to approximately 1,200 subscribers; Project Africa for Rectal Microbicide, a forum providing information regarding rectal microbicide research and development to 53 subscribers; and the Journalists Against AIDS listserv, a listserv distributed to over 2,000 persons and groups participating in the HIV response. The online survey was conducted for 3 weeks.

A total 70 responses comprising 36 (51.4%) men, 31 (44.3%) women, and three (4.3%) participants of unidentified gender were received from the online survey. Only 63 (95.5%) respondents indicated their age as follows: two (2.9%) participants were aged 20 to 24 years; 23 (32.9%) were 25 years to 35 years; 21 (30.0%) were 35 years to 44 years; 16 (22.9%) were 45 years to 64 years; and five (7.1%) were aged 55 years to 64 years. Respondents were from France, Nigeria, the United States, Australia, Congo, Canada, Ghana, and Ethiopia. They included persons working in academia, NGOs, CBOs, faith-based organizations, donor agencies, bilateral and multilateral partners, and the private sector. Fifteen (21.4%) respondents did not work in the HIV response sector. The data were automatically summarized and compiled by the Google Survey tool.

#### Consultative workshops

Nine serodiscordant couples from Cross River, Benue, Abuja, and Edo States, and four representatives from civil society organizations who were living with HIV participated in the consultative workshop. The meeting was conducted in English and Pidgin English because of the low literacy levels of some participants. The objectives of the meeting were to identify barriers, challenges, and facilitators in implementing PrEP for serodiscordant couples; develop strategies to address each barrier and challenge; and cultivate strategies to educate peers on daily PrEP use, including referring peers for screening tests to determine if they qualify for daily PrEP and encouraging compliance with quarterly safety and HIV tests. Participants were divided into groups according to their respective states in order to discuss state-specific peculiarities. Group discussions were guided by a discussion guide, and the same guide was used by all groups. Discussions were then presented to the entire group for further deliberation.

The second meeting included 39 policy makers, health care providers, and representatives of the donor and implementing community in Nigeria. The discussion focused on methods to integrate PrEP into existing health services and facilities, recommendations on ensuring PrEP access by the PrEP demonstration project study population, and staffing needs. Participants were divided into three groups to discuss how PrEP delivery could be integrated with other health services and facilities, facilitation of PrEP access, and staffing issues for PrEP service delivery. Discussions were then presented to the entire group for further deliberation.

### Data analysis

The qualitative data included the following: transcripts from telephone interviews, audio recorded IDI, and FGDs; summary of the online survey results; and hand-written notes (brief field notes, summary notes, debriefing reports) from telephone interviews, IDIs, FGDs, and consultative meetings. Data were inductively examined using a content analytic approach to construct descriptive categories. Responses corresponding to each study objective were summarized and assigned to the descriptive categories, and the categories were further examined to identify general themes and broad concepts. The analytic process began by completely reading the field notes and transcripts to identify content related to the study objectives. Relevant quotes were retrieved from transcripts. Results were compared and linkages made between these various data sources allowing for confirmation, corroboration, and validation of study results through a triangulation process. Findings across sites were compared, and differences or similarities in the responses were explored from various social, economic, and geographical contexts.

### Ethical approval

This study was approved by the Nigerian Institute of Medical Research Health Research Ethics Committee. The study was conducted in full compliance with the approved protocol. All staff, researchers, and field workers engaged in this study were trained on research ethics emphasizing the importance of informed consent and confidentiality. No names or personal identifiers were recorded on any study instruments. All study-related information was stored securely and centrally at the NACA.

## Results

### Appropriate target groups for a PrEP demonstration project in Nigeria

Suggestions on appropriate target populations for the proposed PrEP demonstration project in Nigeria were elicited through the telephone interviews, IDI, and online survey.

#### Telephone interviews

Forty-three (38.1%) respondents in the telephone interview prioritized HIV serodiscordant couples for PrEP access in the proposed demonstration project. One reason cited for prioritizing serodiscordant couples was because HIV prevention for the HIV-negative partner through condom use was considered a barrier to procreation; PrEP would assuage this concern while reducing the risk of HIV infection. Several respondents noted these concerns in the telephone interviews as follows:

*“In marriage where one partner is HIV+ and the other is not, there is no way we can say they should not have sex. We already know the problems we have with condom use, because it is perceived as a sort of barrier in sexual acts. Women often have to negotiate with their partners when they are the ones without the virus. PrEP is actually easier to use for them because it can just be taken before meeting with their partners. It is also likely to give a sense of responsibility on the part of the HIV+ male partners”.****(Telephone interview respondent)***

*“It will give the serodiscordant couples ‘hope’ and increase the level of people disclosing status. And probably improve adherence”. (****Telephone interview respondent****)*

Eighteen (15.9%) respondents in the telephone interview prioritized key affected populations, often referred to as the most-at-risk populations (MARPs) in the national HIV response in Nigeria, for access to PrEP in the proposed for the demonstration project. Reasons cited for prioritizing MARPs included the need to target a small defined population in the pilot study and national epidemiological data showing that 10% of new infections occur in MARPs. This was stated by two respondents as follows:

*“For the MARPs, especially the FSWs, it can be of great help. They can use it 24 hours before sexual acts and it can help limit the prevalence”.****(Telephone interview respondent)***

*“If PrEP is adopted, MARPS should be the target community; a small population is advisable in a pilot study”. (****Telephone interview respondent)***

#### Individual in-depth interviews

Of 111 total IDI participants, 106 suggested specific populations; the majority of respondents (56%) recommended serodiscordant couples as the target population for the proposed PrEP demonstration study. As stated by two interviewees:

*“I think the first group for me [is] the partners of those who are infected with HIV, in other words the serodiscordant couples”. (****IDI respondent****)*

*“For one, I think the population we should think about [target] is the high-risk groups, especially the discordant partners”. (****IDI respondent****)*

One reason given for prioritizing PrEP access to this population was that serodiscordant couples were considered at high risk of HIV infection. Another reason was the ease in working with serodiscordant couples, which helps address the challenges with adherence to the PrEP regimen. Several respondents expressed this concern during the extended interviews:

*“We find HIV serodiscordance among partners of those who are HIV-positive is quite high; I cannot give specific data but I would say it is considerably high”. (****IDI respondent****)*

*“…married people are more reasonable and stable, unlike the youths”. (****IDI respondent****)*

*“If we can get people to embrace it, like the discordant partners, that is a group I think we should expect good success with because they are committed and they are together and they know after counselling that they will really need this drug to stay protected, so they will show strong commitment”. (****IDI respondent****)*

### Online survey

Sixty-one (92.4%) online survey respondents prioritised the use of PrEP by serodiscordant couples, and 29 (43.9%) their enrolment in the PrEP demonstration study. HIV-negative partners in serodiscordant relationships were considered to be at high risk for HIV infection, a risk that could be reduced by PrEP use. The following quotes represent some of the reasons provided:

*“The serodiscordant couples are already living with a HIV-positive partner and are at high risk of contacting the HIV virus”. (***Online survey respondent***)*

*“PrEP will help in mutual adherence for both partners and the success can be predicted”.***(Online survey respondent)**

#### Male and female sex workers

Male and female sex workers received the second highest consideration for PrEP access. Fifty-two (78.8%) online survey respondents endorsed access to PrEP for male and female sex workers, and 15 (22.7%) respondents prioritized their enrolment for the proposed PrEP demonstration project. However, recruiting male and female sex workers for the proposed PrEP demonstration study may prove challenging owing to poor retention caused by their high mobility.

#### Men who have sex with men

Forty-nine (74.2%) online survey respondents endorsed PrEP access in MSM, but only seven (10.6%) respondents prioritized their enrolment in the PrEP demonstration project. In general, respondents felt that MSM should not be prioritized for PrEP access because the social and cultural environment in Nigeria stigmatises and does not support MSM, despite their heightened risk for HIV infection. One reason identified for prioritizing access to PrEP by MSM was the need to achieve the national goal of preventing new HIV infection irrespective of sexual orientation. However, the criminalization of same sex practices in Nigeria makes PrEP access by MSM challenging, as stated by one respondent:

*“As an epidemiological group, MSM face higher risks than others. The political climate in Nigeria, with the recent anti-gay law, might however be a problem for targeting MSM in research, and it is important that research does not increase risk of violence or stigma. For this reason MSM might need to be targeted using a euphemism, such as “men at increased risk of HIV” and allowed to self-identify as “high risk” in that way rather than as an MSM”.***(Online survey respondent)**

#### People who inject drugs

Forty-six (69.7%) respondents on the online survey endorsed PrEP access by PWID, but only one (1.5%) respondent prioritized their enrolment in the PrEP demonstration project. Concerns raised included the need to provide clean needles for study participants, which was considered to encourage intravenous drug use. Also, the PWID population in Nigeria is considerably smaller than other populations at high risk for HIV, such as serodiscordant couples, male and female sex workers, and MSM. This preference was expressed by one respondent as follows:

*“I would still rate them after discordant couples, MSM, sex workers because the percentage of PWID in Nigeria is not as high as the other rates”.***(*Online survey respondent)***

#### Young women aged 21–24 years

Forty-five (68.2%) online survey respondents endorsed young women’s access to PrEP, and 11 (13.6%) prioritized their enrolment in the PrEP demonstration project. Reasons cited for their consideration for study enrolment included being sexually active, being at high risk for HIV infection, their vulnerability to sexual abuse, and their marginal ability to negotiate condom use. This concern was raised by one respondent as follows:

*“More women are HIV-positive in Nigeria. Many women sell sex for family up -keeps, hence it is imperative that this group be considered*. **(Online survey respondent)**

#### Other target populations

Other target populations for a PrEP demonstration project in Nigeria identified through the telephone interviews and IDI were health care workers, uniformed service men, and residents of states with a high HIV prevalence. One respondent recommended individuals in regions facing conflict as a target population as follows:

*“The focus should be especially in the southeast, south, southwest and north-central geopolitical zones of the country where, the incidence of rape and internal displacement is high. People are more vulnerable as a result of rape.” (****Telephone interview respondent)***

### Community interest and perception about PrEP as a HIV-prevention tool

The level of interest and support for PrEP in the general community as perceived by the respondents was discussed in the telephone interviews, IDI, and online survey.

Eighty-four (74.3%) of the telephone interview respondents and 62 (93.9%) of the online survey respondents felt that the PrEP use would provide additional benefits for the country’s HIV response programme by helping to reduce the HIV incidence, increase lifespan, and preserve long-term relationships in serodiscordant couples. Other potential gains included growth of HIV prevention programmes in key affected populations and a greater range of available HIV-prevention options, as stated by several survey respondents:

*“PrEP will provide most men having sex with men (MSM) who do not use any prevention method and who also have heterosexual sex with others in the general population some protection”.****(Online survey respondent)***

*“PrEP will afford serodiscordant couples a new beauty to life as they will be able to live a reproductive life, protect unborn children and have peace of mind”.****(Online survey respondent)***

“*PrEP offers a potential advantage over male and female condom use, which requires partner consent*”. ***(Online survey respondent)***

However, respondents noted that a national PrEP programme will only be successful if the government can ensure its sustained access. Concerns about the level of poverty, heavy workload for health personnel, and the effect of these factors on the efficacy of a proposed PrEP programme were expressed; the success of the PrEP programme therefore, seemed daunting. To ensure the sustainability of a PrEP programme, the Nigerian government must develop a plan that addresses the capacity needs of the health workforce, creates awareness of the program in the community, avoids depending solely on development partners for programme implementation, and invests in the local manufacture of Truvada (a PrEP drug) to reduce its cost. Integrating PrEP into the current HIV-prevention programme and decentralizing PrEP delivery to improve access and coverage are paramount.

Respondents identified several potential community concerns about PrEP. There was overwhelming concern that religious organizations might be unsupportive because promoting PrEP would be seen as promoting sexual promiscuity. However, most respondents believed that with adequate information and sensitization, this challenge could be addressed.

Other concerns expressed included a likely increase in risky sexual behaviours, misconceptions and misinterpretations about PrEP use because of low literacy levels, stigma associated with PrEP use, poor adherence with daily dosing by healthy individuals, and the fear of side-effects potentially decreasing adherence to the PrEP regimen. Respondents stated that:

*“We even have to be careful on how we are going to implement it so that people don’t abuse it. People may just be having sex without condoms and will take Truvada instead … We don’t want PrEP to be abused the way emergency contraception such as “Postinol” have been abused”.****(Telephone interview respondent)***

“*[to be] seen taking drugs all the time could cause fear and stigma” and “people might label them for going to collect HIV drugs”.****(IDI study participants)***

Community leaders and members need to be informed of and involved in the design and implementation of the PrEP demonstration project, which would promote acceptance and ownership of the programme by communities. This requires giving appropriate and clear information about PrEP to communities at dialogue sessions, and through mass public education and sensitization using mass media. Many (53.0%) online survey respondents did not anticipate any major challenges in engaging community leaders in a dialogue about PrEP. In Cross River and Edo States, IDI respondents noted that enactment of a local anti-stigma law would limit stigma and discrimination.

### Communicating about PrEP with different stakeholders

Various stakeholders identified the need to effectively engage the electronic and print media in public education about PrEP. Prime time periods on electronic media could be used to disseminate information. Respondents from the Abuja metropolis recommended using social media such as Facebook, blogs, and Twitter to create awareness about PrEP among young people. Some suggested using euphemistic names such as “Touch and Go”, “Fame”, “Family Vitamins”, and “Hope” for PrEP because of the potential stigma that may be associated with PrEP use.

### Concerns about PrEP use by serodiscordant couples

Stigma was identified as a major concern and potential barrier for PrEP acceptance and use by serodiscordant couples. Identification as someone needing PrEP and the need for repeated hospital visits were considered stigmatizing and suggestive of HIV infection. Other concerns raised were: challenges in taking drugs when not ill, fear of blood samples being used for nefarious purposes, general apathy towards medical examinations, side-effects from ARV therapy use, potential for drug resistance arising from drug unavailability and nonadherence, increased HIV risk behaviour, coping with frequent hospital visits for blood tests, drug refills, and counselling, and the long-term sustainability of the programme.

Serodiscordant couples also identified the need for the proposed PrEP demonstration project to accommodate the social context and realities of their lives. Typically, the index partner is an HIV-positive woman diagnosed during pregnancy. A diagnosis of HIV serodiscordance often leads to a breakdown of the marital relationship. The PrEP programme would therefore need to provide counselling and guidance to beneficiaries. These sessions could address marital issues among others. Support groups for serodiscordant couples would be a veritable means to address this potential challenge.

Innovative HIV testing strategies are also required in order to recruit couples comprising an HIV-negative woman and HIV-positive man. Incentives for HIV testing may promote agreement to testing by men.

One participant in the consultative meeting with serodiscordant couples noted a potential challenge that may arise in encouraging PrEP use, especially by HIV-negative men in serodiscordant relationships, namely that the HIV-negative partner may balk at the PrEP regimen due to their partner’s infection, as expressed in through the response: “*Why should I take drugs for your infection?*” There was also concern on the poor attitude of health care workers towards PrEP users, including breaching confidentiality and stigma expressed by health care workers.

### Suggested design and implementation of a PrEP demonstration project

Suggestions for the design and implementation of a PrEP demonstration project in Nigeria were elicited through the FGDs and consultative meetings. Respondents recommended that PrEP be prescribed free of charge. One reason cited was the need to forestall the sale of counterfeit drugs. However, if drugs must be sold, the suggested price ranged between 50 and 2,000 naira per prescription.

Another concern raised by a respondent on the online survey was that the Nigeria HIV programme will face two different markets of circulation: one (private) for condoms and another (social) for PrEP. A plan is needed to contain these multiple programmes including the development of new HIV-prevention campaigns.

Respondents during the FGD recommended that PrEP drugs be prescribed and dispensed by qualified health workers, particularly doctors, pharmacists, and pharmacy assistants, in both public and private health facilities.

Policy makers and programmers at the consultative meeting for policy makers concurred on the need to decentralize service delivery for PrEP; baseline HIV testing and initial PrEP treatment could be done at tertiary and secondary health facilities, while drugs could be dispensed at primary health centres. In addition, respondents recommended that PrEP services be centralized to the initiating tertiary/secondary facility for the first three months, which is a critical period in the care programme. Laboratory evaluations should also be performed every 6 months at the initiating facility. Drug refills and three-monthly HIV Counselling and Testing should be conducted at private hospitals and primary health care centres linked to the initiating facilities, also referred to as satellite centres. All health care workers at the central and satellite centres should be regularly trained on adherence counselling and ethical health care delivery.

State-specific service delivery models were identified during the consultative meetings for serodiscordant couples and policy makers. For Cross-River State, the serodiscordant couples preferred that PrEP delivery be integrated into routine outpatient care. This, they opined, should reduce potential stigma. In Benue and Edo States however, serodiscordant couples preferred that PrEP delivery be integrated into existing ART programme in order to increase the opportunities for spouses receiving highly-active antiretroviral therapy to pick up PrEP refills for their HIV-negative spouses.

## Discussion

This study shows that there is high public interest and support for the use of PrEP as part of the HIV-prevention armamentarium in Nigeria. The respondents prioritized HIV serodiscordant couples for PrEP access in a planned PrEP demonstration project in Nigeria for several reasons including the challenges faced by couples in continual condom use, the increased prospect for procreation, and the enhanced ability to reduce marital discord. However, several challenges were identified for PrEP use and access, especially the potential for stigma associated with ARV use and the increased likelihood of index partners being women, as most serodiscordant couples are identified through screening for HIV infection in women attending antenatal clinics. For a male dominated society such as Nigeria, this has significant implications for the uptake and use of PrEP by HIV-negative male partners in serodiscordant relationships.

The design of an HIV-prevention package that includes PrEP for HIV serodiscordant couples in Nigeria is apt. A high incidence rate of 1.2 per 100 person-years among serodiscordant couples was reported in a highly controlled clinical trial where aggressive viral suppression was provided for the index partner [17]. Approximately 20% of seroconversion events in serodiscordant couples are caused by a sexual partner other than the index partner [18]. For many couples, consistent condom use can be challenging especially in a culture where having children is of great importance [18]. Several reports show that serodiscordant couples increasingly have multiple sexual partners over life and often indulge in unprotected sexual intercourse [19]. Unprotected sex may also occur in an attempt to conceive, further increasing the risk for seroconversion even when the sexual partner is virologically suppressed [20].

Stigma was identified as a potential barrier for PrEP use and discrimination a potential consequence of its use. This is likely a reflection ofs the stigma associated with ARV use for management of HIV infection. Stigma has long been identified as a deterrent for uptake of HCT services [21], agreement to antiretroviral therapy [22], and adherence to the ARV drug regimen [23]. Education about the possible use of ART for HIV prevention may broaden the public’s knowledge and understanding of PrEP. However, the potential for public education to stem reluctance in communities where ART is stigmatised may be limited. The potential for stigma associated with PrEP use has been previously identified [24,25] and is a real concern in people using PrEP [24]. It is a recognized cause of low adherence and discontinuation of therapy [26]. The PrEP demonstration project should explore strategies for communicating about PrEP in ways that do not perpetuate stigma. For example, the PrEP demonstration project could be open to all persons who perceive themselves at high risk of HIV infection. Recruitment can then be limited to those at highest risk for HIV infection, especially those who have HIV-positive sex partners. PrEP users must be empowered to prevent stigma from family, friends, and significant others. Information about PrEP should also be included in stigma reduction campaigns [26,27]. Public education programmes should enable people to assess their beliefs and address the underlying prejudices that may affect decision making and actions.

Beyond stigma, the feasibility of covert use of PrEP by women may also be limited by the cultural contexts that define male–female relationships. Many persons in Africa do not support covert use of PrEP products (whether pills, gel, or a potential injectable), except perhaps with casual or paying partners. While this study did not explore the feasibility of covert use of pills as PrEP for HIV prevention by women, it is important that this consideration be factored into the design and implementation of the PrEP demonstration programme.

A PrEP study for serodiscordant couples requires that couples know their HIV status. Where individuals disclose their HIV-positive status to their partner, partner support is a well-known motivator for ART adherence [28,29]. However, many individuals never disclose their HIV status to their partners [29] for fear of partner violence, stigma, and loss of economic support, among other reasons [29]. Oftentimes, serodiscordance is identified in the antenatal clinic during couple testing. This implies that the majority of participants in a PrEP demonstration project will be men. In a male dominated society such as Nigeria, HIV-negative male partners who use PrEP may face stigma owing to HIV infection of the female partner. Many participants may balk at facing this stigma or taking medication owing to their partner’s infection. The reality of this challenge moves the discussion of PrEP access and use in serodiscordant relationships well beyond the biomedical sphere. The realities of individuals living with HIV and the challenges of serodiscordance must be considered in the design and implementation of PrEP access programmes. Marital counselling may therefore be required in the design of the PrEP demonstration project as highlighted by the respondents in the consultative workshops.

There were concerns raised about resistance resulting from drug unavailability and nonadherence to therapy. The development of resistance to ARVs used as PrEP is possible. In trials where monitoring and follow-up of participants is strict and regimented, the number of patients who developed resistance to PrEP medication was extremely low; two participants enrolled in an iPrEx study with undetected, seronegative acute HIV infection who were randomized to receive daily oral tenofovir-emtricitabine developed resistance to emtricitabine [30]. A similar case was also reported in the TDF2 study [30]. When HIV-positive individuals do not adhere to therapy, resistance may arise and this is a challenge. However, the potential for resistance in PrEP is more complex, as poor compliance would more likely lead to HIV infection [28]. Increased exposure to partially suppressive ARVs raises the risk of selecting resistance mutation, which may compromise the preferred regimen should the individual seroconvert. Furthermore, it may impact the efficacy of a second-line antiretroviral therapy regimen in Nigeria [28]. At a population level, PrEP could lead to drug resistance and limit access to ARV beyond the second-line regimen [30,31]. The National PrEP demonstration project should consider the immediate and long-term implications of participants developing resistance to the PrEP regimen and strategies to reduce or eliminate the potential for resistance during real life access.

The concern expressed over revamping the HIV-prevention campaign is real. Most recently, the US Centers for Disease Control and Prevention, in response to the campaign by the HIV Prevention Justice Alliance, agreed with the need to avoid the term “unprotected sex” and instead specifically refer to condom use. This is critical because a person on ART who is virologically suppressed can actually have safe sex even without using a condom [32]. The cost associated with revising documents and educational campaigns may be enormous, but is necessary in light of emerging scientific and pragmatic evidence. The national PrEP demonstration project will need to assess the impact of these public educational messages on the general understanding of ARV use in reducing the risk for sexual transmission of HIV.

While HIV serodiscordant couples are at high risk for HIV infection, especially in Nigeria where child conception following marital union is a priority, there are other populations in Nigeria who are also at high risk of HIV infection, as the present study acknowledges. We however cannot readily identify reasons why the access of HIV serodiscordant couples to PrEP as prioritized over access to PrEP by other high risk populations. Several responses alluded to the unsupportive legislative environment that makes working with MARPs challenging. The lessons learned during implementation of the current national HIV response programme for key affected populations in Nigeria [33,34] will inform the design of a PrEP access programme for MSM, FSW, and PWID as it is scaled up to ensure wider PrEP access to multiple populations in Nigeria.

## Conclusion

It is clear that there will be challenges associated with the acceptance of PrEP by HIV serodiscordant couples in Nigeria, but many of the challenges identified are surmountable. However, much depends on the actions taken by multiple players including the capacity to conduct couple counselling and identify serodiscordant couples, motivation of HIV-negative male partners to use PrEP, adherence to the PrEP regimen and HIV monitoring, and effective public education addressing stigma. Concerns about the risk of increased resistance and the potential loss of principal treatment options should be seriously considered. Therefore, as one of its goals, the PrEP demonstration project in Nigeria should determine which HIV serodiscordant couples are most likely to benefit from the intervention and most likely to comply with a regimented care plan incorporating both PrEP and frequent HIV testing [20]. It should also identify how best to optimize drug adherence among PrEP recipients and intervene if the drugs are not taken properly [31].

## Abbreviations

AIDS: Acquired Immune Deficiency Virus
ART: Antiretroviral therapy
ARV: Antiretrovirals
CBO: Community Based Organisation
FGD: Focus Group Discussions
FSW: Female Sex Worker
HCT: HIV Counselling and Testing
HIV: Human immunodeficiency virus
IDI: In-Depth Interview
MSM: Men who have sex with Men
NACA: National Agency for the Control of AIDS
NGO: Non-Governmental Organisation
PrEP: Pre-exposure prophylaxis
PWID: People who Inject Drugs
